# Unintentional falls among children in rural Ghana and associated factors: a cluster-randomized, population-based household survey

**DOI:** 10.11604/pamj.2021.38.401.28313

**Published:** 2021-04-26

**Authors:** Adam Gyedu, Godfred Boakye, Robert Quansah, Peter Donkor, Charles Mock

**Affiliations:** 1 Department of Surgery, School of Medicine and Dentistry, Kwame Nkrumah University of Science and Technology, Kumasi, Ghana,; 2 University Hospital, Kwame Nkrumah University of Science and Technology, Kumasi, Ghana,; 3 Ghana Armed Forces, Accra, Ghana,; 4 Department of Surgery, University of Washington, Seattle, WA, USA,; 5 Global Injury Control Section, Harborview Injury Prevention and Research Center, Seattle, WA, USA

**Keywords:** Household falls, child, rural, household, Ghana

## Abstract

**Introduction:**

falls contribute to almost one-fifth of injury-related deaths. The majority of these occur in low- and middle-income countries. The impact of fall injury in low- and middle-income countries is greater in younger individuals. We aimed to determine the epidemiology of falls among rural Ghanaian children.

**Methods:**

from March to May, 2018, we conducted a cluster-randomized household survey of caregivers in a rural Ghanaian sub-district, regarding household child falls and their severity. We utilized a previously validated survey tool for household child injury. Associations between household child falls and previously described predictors of household child injury were examined with multivariable logistic regression. These included age and gender of the child, household socioeconomic status, caregiver education, employment status, and their beliefs on why household child injuries occur.

**Results:**

three hundred and fifty-seven caregivers of 1,016 children were surveyed. One hundred and sixty-four children under 18 years had sustained a household fall within the past six months, giving a household child fall prevalence of 16% (95% C.I, 14%-19%). Mean age was 4.4 years; 59% were males. Ground level falls were more common (80%). Severity was mostly moderate (86%). Most caregivers believed household child injuries occurred due to lack of supervision (85%) or unsafe environment (75%); only 2% believed it occurred because of fate. Girls had reduced odds of household falls (adjusted O.R 0.6; 95% C.I 0.4-0.9). Five to nine year-old and 15-17 year-old children had reduced odds of household falls (adjusted O.R 0.4; 95% C.I 0.2-0.7 and 0.1; 95% C.I 0.02-0.3, respectively) compared to 1-4 year-olds. Caregiver engagement in non-salary paying work was associated with increased odds of household child falls (adjusted O.R 2.2; 95% C.I 1.0-4.7) compared to unemployed caregivers. There was no association between household child falls and caregiver education, socioeconomic status and beliefs about why household child injuries occurred.

**Conclusion:**

the prevalence of household child falls in rural Ghana was 16%. This study confirms the need to improve supervision of all children to reduce household falls, especially younger children and particularly boys. Majority of caregivers also acknowledge the role of improper child supervision and unsafe environments in household child falls. These beliefs should be reinforced and emphasized in campaigns to prevent household child falls in rural communities.

## Introduction

Injury continues to be a major global public health problem. It claims about 4.3 million lives and accounts for 10% of all disability-adjusted life years incurred annually [1]. Falls are responsible for 17.5% of all injury-related deaths annually, with 35,000 involving individuals below 20 years [1]. The contribution of falls as a leading cause of death is expected to increase by 2030 [2]. The majority (71%) of fall injuries occur in low- and middle-income countries (LMICs) which are the least equipped to provide proper trauma care [1,3]. In Mozambique, falls were the leading cause of injuries among children (0 - 14 years) reporting to public referral hospitals in Maputo [4]. Likewise, falls were the most common injury type among children under 15 years in Sudan, representing 30% of all childhood injuries recorded in a community survey [5].

The impact of injury in LMICs appear to be greater in younger individuals. Ninety-five percent of all injury-related deaths among individuals under 20 years occur in those living in LMICs. Additionally, 97% of all injury-deaths caused by falls in this age group occurs in LMICs [1]. Nonetheless, the burden of childhood falls is largely accounted for by the morbidity and disabilities that may persist for life. Most falls experienced by children are reported to occur in or around the home environment [6], and majority of these may be regarded as being a normal part of their development. However, the curiosity to explore their surroundings is characteristically not matched by their ability to assess danger or respond appropriately [7]. As children grow older and increasingly independent, they have access to more space and are capable of a broader range of physical activity. With this increased access and capability, they also tend to engage in more risk-taking behavior.

A previous assessment identified falls as the most common mechanism of household child injuries in semi-urban Ghanaian communities, contributing to more than half of all injuries [8]. Several LMIC studies have found associations between child characteristics (age and gender), caregiver characteristics (including education, employment, and perceptions on why household child injuries occur) and household child injuries [6,8-11]. The contribution of falls as a mechanism of household child injuries in LMIC rural communities has not been well-studied. Thus, we sought to determine the prevalence of falls as a cause of household child injuries in rural communities in Ghana and factors associated with higher prevalence of falls. By doing so, the findings could inform effective fall prevention interventions among children living in such communities.

## Methods

**Study population:** Ghana is a lower middle-income country with a population of 30 million; about 45% live in rural communities [12]. Many rural communities are close to more populated areas with resources such as health promotion initiatives and better-equipped health facilities. Inhabitants of most rural communities in Ghana that are close to more populated areas are often engaged in subsistence activities like farming or petty trading or work in low-wage jobs within the adjacent urban area [13]. Our study was conducted in Amakom, a rural sub-district in the Ashanti region of Ghana. Amakom had a 2018 estimated population of 18,988, 74% of whom were children under 18 years with 3,779 children under five years [14]. We assumed the 4% injury prevalence reported among children below 5 years in rural Pakistan [15], 95% confidence level, 10% error margin and a design effect of 2, to estimate a sample size of 738 [16].

**Survey design:** we conducted a cluster-randomized, population-based, household survey, in March - May 2018, to examine falls occurring among children in and around the house in rural communities close to large metropolises. Details of the sampling methodology have previously been described [13] and are summarized herein. First, computer random sampling was used to select one of six rural districts bordering the Kumasi metropolis in Ghana (Bosomtwe). Second, one of Bosomtwe's three sub-districts, which are similar in demographics, was randomly selected (Amakom). Third, 6 of 11 community clusters in Amakom were randomly selected. We visited every dwelling within each selected community. Dwellings were often comprised of multiple households. However, only households with a child under 5 years were eligible for the survey. We randomly selected one eligible household within each dwelling. Thus, our sample was representative of households with caregivers of children under 5 years in Amakom. Using a previously validated structured questionnaire [8,17], the primary caregiver of a child under five years within selected households was interviewed about household child falls. A household child fall was defined as a fall sustained by a child below 18 years under care of the particular caregiver within the past six months that was sustained inside and up to 200 meters from the house. Multiple falls sustained by a child were counted once. The severity of the falls were classified according to previously described definitions as follows [18]: moderate - resulted in the child missing at least 1 day of school or work or seeking healthcare without being hospitalized; major - resulted in the child being hospitalized for 1 to 9 days; serious - resulted in the child being hospitalized for 10 or more days; severe - resulted in permanent disability; fatal - resulted in death. Local enumerators administered the survey in the predominant language spoken in the study area (Twi). These were the same university graduates employed for data collection in the previous study [8]. They were trained on the wording of the questionnaire to maintain consistency of translation from English to Twi and back-translation to English. Enumerators collected demographic and household characteristics on ownership of consumable goods and physical characteristics of the household, which were used to create a wealth index using principal component analysis (PCA). PCA scores were divided into quintiles, the first quintile representing the poorest households. They also collected demographic information of children under 18 within the selected household and characteristics of falls they had sustained within the past 6 months. Finally, caregivers were asked why they thought household child injuries occurred. The data were checked for quality by re-administering the questionnaire to a randomly selected 10% of caregivers. All survey respondents provided informed consent. The Kwame Nkrumah University of Science and Technology Committee for Human Research, Publications and Ethics approved the study (protocol number: CHRPE/AP/589/17).

**Data analysis:** data were entered using the open data kit application [19]. Analyses utilized survey sampling weights, adjusted for clustering by community and household. Community weights (Wc) were determined by dividing the total number of caregivers selected (357) by the number caregivers selected from each community. Household weights (Wh) were determined as the inverse of the probability of selecting a particular caregiver from the number of eligible caregivers within a dwelling. The sampling weight applied to each caregiver was thus Wc X Wh. All analyses were done using survey weights in STATA version 14 (StataCorp. LP, College Station, USA). Data were expressed as descriptive statistics. Multi-level, mixed-effects logistic regression was used to examine the association between the occurrence of household child falls and several covariates. We included a priori selected variables of interest which have been previously described as potential predictors of household child injury in LMICs such as age and gender of the child, household socioeconomic status, caregiver education, employment status, and their beliefs on why household child injuries occur [6,8-11]. Caregiver education was dichotomized as “no formal education” and “any level of formal education”. Household socioeconomic status was considered as a continuous variable. There was no collinearity between variables and there was a normal distribution of deviated residuals from the model. P-values <0.05 were considered significant.

## Results

**Household characteristics:** there were 715 dwellings within the six selected communities in Amakom; 358 of them contained 567 households with eligible caregivers. Three hundred and fifty-eight eligible caregivers were randomly selected, one caregiver per dwelling. Only one eligible caregiver did not participate due to consistent unavailability. Thus, we surveyed 357 individuals who represented 2,713 caregivers in Amakom [13]. Twenty-nine percent (95% C.I 24% - 35%) of caregivers responded to having children who had sustained a household fall within the past six months. Mean caregiver age was 34 years. They were mostly female (92%) and 74% were mothers to the children. The majority (70%) of them had only basic education and engaged in non-salary paying work (83%). Three-quarters of them lived in their own accommodation. A large proportion of them believed household child injuries occurred due to lack of supervision (85%) or unsafe environment (75%), while very few (2%) believed it occurred because of fate (Table 1).

**Table 1:** characteristics of children sustaining household falls in Amakom sub-district, Ghana

	Unweighted		Weighted	
	%	Frequency (n=164)	%	95% CI
**Caregiver characteristics**				
Age (mean, range)	34	(16 - 85)	34	
Female	88		92	82 - 93
**Relationship to child**				
Mother	76	125	74	69 - 82
Father	9	15	7	6 - 15
Other^a^	15	24	19	10 - 21
**Education**				
None	19	31	23	14 - 26
Basic	71	117	70	64 - 78
Senior high school	10	16	7	6 - 15
Tertiary	0	0	0	-
**Accommodation**				
Rented room or flat	26	42	25	19 - 33
Owner occupier (completed building)	60	99	62	52 - 68
Owner occupier (uncompleted building)	14	23	13	9 - 20
**Employment status**				
Unemployed	21	34	17	15 - 28
Non-salaried worker	79	130	83	72 - 89
Salaried worker	0	0	0	0
**Socioeconomic status**				
1 (Lowest quintile)	20	33	24	15 - 28
2	19	31	19	14 - 27
3	15	24	14	10 - 22
4	20	32	21	15 - 27
5 (Highest quintile)	23	37	20	18 - 31
Missing ^b^	4	7	-	-
**Beliefs on why injuries occur**				
Due to fate	1	3	2	0.5 - 6
Lack of proper supervision	84	138	85	80 - 85
Due to unsafe environment	68	111	75	69 - 75
**Child characteristics**				
**Age of child**				
1 - 4	66	108	68	58 - 72
5 - 9	24	39	21	18 - 31
10 - 14	9	14	10	5 - 14
15 - 17	2	3	1	0.6 - 6
**Sex**				
Male	54	89	59	47 - 62
Female	46	75	41	38 - 53
**Cause of fall**				
Ground level fall	79	129	80	72 - 84
Fall from height	20	33	20	15 - 27
Fell into pit	1.2	1	0.9	0.3 - 4.8
**Injury severity** ^c^				
Moderate	87	143	86	81 - 92
Major	10	16	12	6 - 15
Serious	2	3	1	0.5 - 6
Severe	1	2	1	0.3 - 5
Fatal	0	0	0	-

a Grandparent, older sibling or aunt; ^b^ Missing socioeconomic status due to absence of ≥1 consumable good within the household; ^c^ Injury severity: moderate - child missed ≥ 1 day of school or work or sought healthcare without being hospitalized; Major - child hospitalized for 1-9 days; Serious - child hospitalized for ≥ 10 days; Severe - resulted in permanent disability; Fatal - resulted in death

**Child characteristics:** there were 1,016 children under 18 years in our sample, representing 14,032 children in Amakom. The mean age of children who had sustained a fall was 4.4 (range: 1 - 17) years. Most of them (68%) were 1 - 4 years old and more than half (59%) were males.

**Prevalence, mechanism and severity of injury:** one hundred and sixty-four children under 18 years had sustained a household fall within the past six months, giving a household child fall prevalence of 16% (95% C.I, 14% - 19%). Falls were the cause of 37% of all household child injuries in Amakom (unpublished). The majority of falls (80%) were ground level falls, involving the child falling from slipping, tripping or running, and 20% were falls from a height. These included falls from a bed (7%), table (4%), tree (3%), or wall (2%). For children aged 1 - 9 years, 15% were falls from heights and 84% were falls from ground level, whereas for children aged 10 - 17 years, 52% were falls from heights and 48% were falls from ground level (p=0.004). Almost 90% of the falls were of moderate severity (i.e. the child missed school or work or sought medical care without being hospitalized) (Table 1). For ground level falls, the severity was moderate (88%), major (11%), and severe (1%) whereas among falls from a height, the severity was moderate (79%), major (16%) and serious (4%) (p=0.02).

**Factors associated with household child falls:** there was evidence for female children having reduced odds of household falls (adjusted O.R 0.6; 95% C.I 0.4 - 0.9). Children whose primary caregivers were engaged in non-salary paying work had increased odds of household falls compared to those whose caregivers were unemployed (adjusted O.R 2.2; 95% C.I 1.0 - 4.7). Compared to 1 - 4 year old children, older children had reduced odds of household falls with increasing age associated with lesser odds of household falls (adjusted O.R 0.4; 95% C.I 0.2 - 0.7 for 5 - 9 year olds and adjusted O.R 0.1; 95% C.I 0.02 - 0.3 for 15 - 17 year old children). There was no association between caregiver beliefs about why household child injuries occurred and household falls among children. Additionally, caregiver education and socioeconomic status was not associated with risk of household child falls (Table 2).

**Table 2:** factors associated with household falls among children in Amakom sub-district, Ghana

	O.R	(95% C.I)	p-value	aOR	(95% C.I)	p-value
**Child sex**						
Male	Referent			Referent		
Female	0.7	0.5 - 1.1	0.09	**0.6**	0.4 - 0.9	0.02
**Child age**						
1 - 4	Referent			Referent		
5 - 9	0.4	0.3 - 0.7	0.001	**0.4**	0.2 - 0.7	<0.001
10 - 14	0.3	0.2 - 0.7	0.002	**0.3**	0.1 - 0.7	0.004
15 - 17	0.1	0.02 - 0.3	<0.001	**0.1**	0.02 - 0.3	<0.001
**Caregiver age**	1	0.97 - 1.01	0.42	0.99	0.97 - 1.02	0.65
**Caregiver education**						
No	Referent			Referent		
Yes ^a^	1.3	0.7 - 2.6	0.79	1.3	0.7 - 2.9	0.38
**Caregiver employment**						
Unemployed	Referent			Referent		
Non-salaried worker	1.5	0.8 - 2.7	0.24	**2.2**	1.0 - 4.7	0.04
**Socioeconomic status** ^b^	1.0	0.8 - 1.1	0.69	1.0	0.9 - 1.2	0.77
**Caregiver believes injury occur due to fate**						
No	Referent			Referent		
Yes	0.6	0.2 - 1.8	0.35	0.7	0.2 - 2.3	0.51
**Caregiver believes injury occur due to lack of adequate supervision**						
No	Referent			Referent		
Yes	1.3	0.6 - 3.0	0.51	1.3	0.5 - 3.3	0.64
**Caregiver believes injury occur due unsafe environment**						
No	Referent			Referent		
Yes	0.9	0.6 - 1.5	0.73	0.9	0.5 - 1.5	0.64

Odds ratio in bold denote those with p <0.05; ^a^ Any level of education; ^b^ Socioeconomic status (wealth quintiles) considered as a continuous variable

## Discussion

Falls are the most common type of injury among children, resulting in over 4 million disability-adjusted life years among individuals under 20 years, over 90 percent of which occur in LMICs [1]. In low socioeconomic communities within these countries, play areas are usually not restricted to playgrounds. Thus the little available space, crowded public places, and sometimes uneven terrain in the community that children use for their recreation make them more vulnerable to falls [20]. Our data suggest a high prevalence of household falls among rural Ghanaian children. However, majority of these were ground level falls that largely resulted in injuries of moderate severity. Female gender and older child age were associated with reduced odds of household child falls. Non-salary paying caregiver jobs were associated with increased odds of household child falls. These data provide opportunities for prevention of household falls among rural children in Ghana and other LMICs. The high prevalence of household child falls we found in this rural community (16%) is similar to the 15.8% prevalence previously reported in a semiurban community in Ghana [8]. However, while falls represented 37% of all household injuries sustained by rural children, they accounted for more than half of injuries reported for the semiurban children. This may be explained by more hazards within the increasingly more complex but largely improperly built environment present in semiurban areas compared to rural communities. Majority of falls occurred at ground level as reported in other studies [21,22]. Most of the household child falls reported by our respondents were only moderate in severity, which may be attributed to the fact that majority of the falls were at ground level. Falls from a height, such as trees, windows, or roofs, usually result in more severe injuries [7,23]. In a Kenyan study of falls from a height among 0 - 13 year old children that required hospital admission, 43% sustained fractures, 25% had central nervous system injuries and 17% sustaining facial injuries [24].

Girls had a 40% reduced odds of household falls in our study. Males have consistently been shown to have increased odds of sustaining falls irrespective of the context of the fall or community of residence [5,8,21,25]. This has been partly explained by patterns of child upbringing, socialization and role expectations. Irrespective of culture, boys naturally engage in more risk-taking behavior than girls. Parental practices also tend to foster greater exploratory behavior among boys while imposing fewer restrictions on them [7]. Older children also had reduced odds of household falls, compared to younger children. Older children are more likely to be aware of their surroundings making them more careful while walking or running. Conversely, there was a trend toward a higher proportion of older children sustaining falls from a height compared to younger children, which may be due to increased tendency of children to engage in risk-taking behavior as they grow older [7]. Children whose caregivers were engaged in non-salary paying work had twice the odds of sustaining household falls compared to children of unemployed caregivers. In an Indian study of unintentional injuries among 1 - 5 year old children, falls accounted for 95% of injuries and children of working mothers had significantly higher odds of injuries on bivariate analysis [26]. Similar results were also reported by a study from Egypt [10]. These findings highlight the important role adequate maternal time allocation might have, not only on household child falls but on household child injuries in general [27]. Despite the lack of evidence for an association between unsafe environment or lack of proper supervision and household child falls from our data, majority of caregivers in our study believed that these contributed to household child falls. This presents opportunities for reinforcing such beliefs among rural caregivers and organizing childhood falls prevention initiatives around these beliefs as their contribution to household child falls is well-documented [4,11,28].

Before drawing conclusions from our study, there are some limitations that need to be acknowledged. First, while this was a population-based randomized household survey, the study sample was not powered specifically for childhood fall injuries and risk-factor sub-groups. Thus, falls resulting in permanent disability or death may be underrepresented. Second, the study is based on responses provided by caregivers, the accuracy of which could not be independently confirmed. Third, there were no reports of falls among infants. This might be a random finding but might also be due to caregivers providing socially-desirable responses, especially if those falls led to very severe outcomes. In LMICs, infants have been reported to be at greatest risk of falls resulting in severe injuries, including death [29,30]. Finally, prior physical or mental disabilities of the child [31], or mental health problems affecting caregivers [32], which could contribute to household child falls, were not explored. Despite these limitations, our study provides valuable insights into household falls among rural Ghanaian children and provides opportunities for reducing the commonest injury type in this important demographic area of Ghana.

## Conclusion

This study highlights the high prevalence of household falls among Ghanaian children living in rural communities. It confirms the need to improve supervision of all children to reduce household falls, especially among younger children and particularly boys. Well-designed playgrounds for children may reduce their exposure to hazards while they play, particularly when caregivers are at work. Majority of caregivers also acknowledge the role of improper child supervision and unsafe environments in household child falls. These beliefs should be reinforced and emphasized in campaigns to prevent household child falls in rural communities.

### What is known about this topic

- The majority of household child falls are ground level falls;
- Most household child falls only result in the child missing school or work or seeking medical care without being hospitalized.

### What this study adds

- There is a high prevalence of household falls among rural Ghanaian children;
- Majority of caregivers of rural Ghanaian children believe that lack of supervision or unsafe environment contribute to household child falls;
- This study confirms the association of gender, age of the child and type of caregiver occupation with household child falls.
